# Community assembly rules affect the diversity of expanding communities

**DOI:** 10.1002/ece3.1251

**Published:** 2014-10-03

**Authors:** Zechen Peng, Shurong Zhou

**Affiliations:** 1 State Key Laboratory of Grassland Agro-ecosystems, School of Life Sciences, Lanzhou UniversityLanzhou, 730000, China; 2 Ministry of Education Key Laboratory for Biodiversity Science and Ecological Engineering, School of Life Sciences, Fudan University2005 Songhu Road, Shanghai, 200438, China

**Keywords:** climate change, community similarity, neutral model, range expansion, species richness, trade-off

## Abstract

Despite centuries of interest in species range limits, few studies have taken a whole community into consideration. Actually, multiple species may simultaneously respond to environmental changes, for example, global warming, leading a series of dynamical communities toward the advancing front. We investigated multiple species range expansions through the analysis of a two-species dispersion model and simulations of multiple species assemblages regulated by neutral and fecundity–survival trade-offs (FSTs), respectively, and found that species assemblages regulated by different mechanisms would initiate different expanding patterns in geographic ranges in response to environmental changes. The neutral model generally predicts a higher biodiversity near the core of an expanding range, and a lower community similarity compared with a FST model. Without considering the evolution of life history traits, an assortment of the reproduction ability happens at the advancing front under FSTs at the expense of a higher death rate or lower competitive ability. These results emphasize the importance of community assembly rules to the biodiversity maintenance of range expanding communities.

## Introduction

A species range is very often highly dynamic, with recurrent expanding, shifting, and contracting in the evolutionary history of many species (Brown et al. 1996; Davis and Shaw 2001). A prevailing idea related to species geographic range is that climate is the key limiting factor (Sexton et al. 2009). However, many studies have demonstrated the importance of species interactions, for example competition, followed in frequency by other biotic factors such as predation and parasitism. which either facilitate or limit species distributions (Pulliam 2000; Briers 2003; Derivera et al. 2005; Alexander et al. 2007; Araújo and Luoto 2007; Pigot and Tobias 2013; Raffa et al. 2013), and the evidence for climatic limitation at distribution edges weakens as we move from studies documenting the presence/absence patterns to those documenting the underlying processes (Sexton et al. 2009). Actually, abiotic and biotic factors may interact to regulate the geographic ranges of a species (Taniguchi and Nakano 2000).

Another common feature of previous studies in terms of species range limits is their focus on how a single or a limited number of species respond to abiotic or biotic environment changes, leaving the other members of the communities into a blind background. This may be partially due to the complexity of a field investigation when a number of species are involved. However, theoretical modeling in a community or food web context is also scare (but see Case et al. 2005). Hence, given the dominant driving abiotic factors, like climate changes, on the one hand, and the species interactions on the other, how species in a community assembled by different rules will expand their specific ranges remain unclear.

At the community level, the biotic and abiotic factors regulating species assemblages within a same trophic level can be summed and translated into different mechanisms (Chesson 2000). For example, the neutral theory of biodiversity has been proposed at the beginning of this century as an alternative to the niche theory to explain species coexistence (Hubbell 2001; Volkov et al. 2003; Etienne 2005; Rosindell and Cornell 2007). Although the neutral model has successfully reproduced several macroecological patterns in real communities, the fundamental ecological equivalence assumption that individuals of different species share the same probability of birth, death, migration, and speciation has met with little empirical support (Chave 2004; Wootton 2005). The only way to reconcile the neutrality assumption of the neutral theory and the species differences found in natural communities is through demographic trade-offs, that is FSTs among different species (Lin et al. 2009). Lin et al. (2009) successfully demonstrated that neutrality can be guaranteed by FSTs in a spatially implicit community. Although it has been criticized that such a demographic trade-off must be elaborately maintained to ensure neutrality and that it is easily susceptible to disturbance, for example a stochastic arrival of seeds (Purves and Turnbull 2010) and demographic trade-offs of this category may minimize fitness difference among species and therefore be able to maintain even a higher diversity than a neutral community (He et al. 2012). However, how biodiversity maintained by trade-offs changes along an expanding gradient in response to environmental changes, for example global warming, remains largely unexplored.

In this study, we explore the consequences of movements of multiple species regulated by neutral and trade-off mechanisms via both theoretical analysis and spatially explicit simulations. Similar to Lin et al. (2009), we model long-lived sessile species such as perennial plants. We compare biodiversity and community similarity under different community assembly rules along the expanded ranges of the species. We also investigate how species traits may be assorted at the advancing front.

## Theoretical analysis of a two-species model

Assume that there are two adjacent sites, with site 1 consisting of two species with an equal initial abundance of *K*/2, where *K* is the maximum community size of each site, and site 2 is initially empty. The mean fecundity of an individual of species *i* per year is *Fb*_*i*_, where *b*_*i*_ lying within (0, 1) is a parameter for the per capita fecundity rate scaled by the constant *F*. Assume that an individual of species *i* may die with the probability of *d*_*i*_ in each year and all the species in a local community obey a perfect FST, which means that all species have the same ratio of per capita fecundity to death rate, that is *Fb*_i_/*d*_i_ is a constant. For the sake of brevity and without incurring a loss of generality, we can simply let *b*_*i*_/*d*_*i*_ = 1. Thus, the dynamics within a local community is spatially implicit and neutral (Lin et al. 2009). Let *α* denotes the fraction of the seeds produced by an adult in site 1 that stay in their natal site, and the remaining fraction (1−*α*) is dispersed onto another site. Clearly, *α* is a measure of the intensity of dispersal limitation. Then the probability that species 1 in site 1 will colonize the vacant site 2 after 1 year, and the relative abundance of species 1 in case of successful colonization is


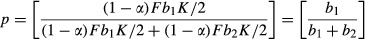


Similarly, the probability for species 2 is *b*_2_ / (*b*_1_ + *b*_2_). The higher a species' fecundity (death) rate, the higher probability that the species will colonize a new site in its range expansion. That is, species with higher fecundity/death rates are more likely to surf on the waves of a range expansion. Contrary to FST, a neutral community (*b*_1_ = *b*_2_) will be solely governed by a random drift, with two species having the same probability to expand their ranges.

## Simulating the dynamics of multiple species assemblages during range expansions: methods

The landscape is assumed to be a two-dimensional regular grid of 25 (rows) × 500 (columns) sites, with each site containing a community up to *K* individuals. Each simulation is initiated in a saturated community located in the middle of the leftmost side of the rectangle (latitude *i* = 13, longitude *j* = 1), within which 20 species share the same initial abundance. Other sites in the landscape are empty at the beginning of each simulation.

The life cycle of individuals in each site per year is modeled as follows: reproduction, death, dispersal, and colonization. The fecundity of an individual of species *i* follows the binomial distribution of (*F*, *b*_*i*_) with the mean fecundity as *Fb*_*i*_, where *b*_*i*_ is randomly drawn from (*c*, 1) representing the per capita fecundity rate scaled by the constant *F*. For the neutral model, we assume *d*_*i*_ = *b*_*i*_ = 0.1, 0.5, and 0.9, respectively, in the simulations.

Death may follow reproduction, and it is assumed that an individual of species *i* may die with the probability of *d*_*i*_ = *b*_*i*_. Then seeds produced disperse according to the dispersal modes. We simulate two kinds of dispersal modes. The first is the nearest neighbor dispersal, with which we assume that proportion 1−*α* of the seeds produced randomly disperse onto eight nearest neighbor sites. For the second type of dispersal, each seed disperses according to a fat-tailed dispersal kernel of


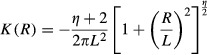


where *K*(*R*) indicates the value of the radially symmetric kernel which is a distance of *R* away from its parent individual, with *η* measuring the fatness of the tails and *L* describing the “width” of the kernel (Chave and Leigh 2002; Rosindell and Cornell 2009). Seeds arriving at a site compete for the vacancies in the community. We ignore those seeds that migrate out of the boundary or to an occupied site.

We repeat the cycle described above and record species richness (number of species), and mean per capita death rate of each community at year A, 2A, 3A, and 4A. The constant A for each parameter set is selected to ensure a clear revealing of the colonization process along the longitude. We also calculate the abundance-based Morisita index among the communities with the same longitude at year A, 2A, 3A, and 4A (Chao et al. 2008).

For each parameter set, 50 independent iterations are performed, and the reported values are averages over communities with the same longitude and over 50 iterations.

In the simulations, we set *F =* 100, *c* = 0.01, *K* = 1000, *η* = −6, *α* = 0.1, 0.5, and 0.9 and *L* = 1, 4, and 16, respectively.

## Simulating the dynamics of multiple species assemblages during range expansion: results

**Figure 1:**
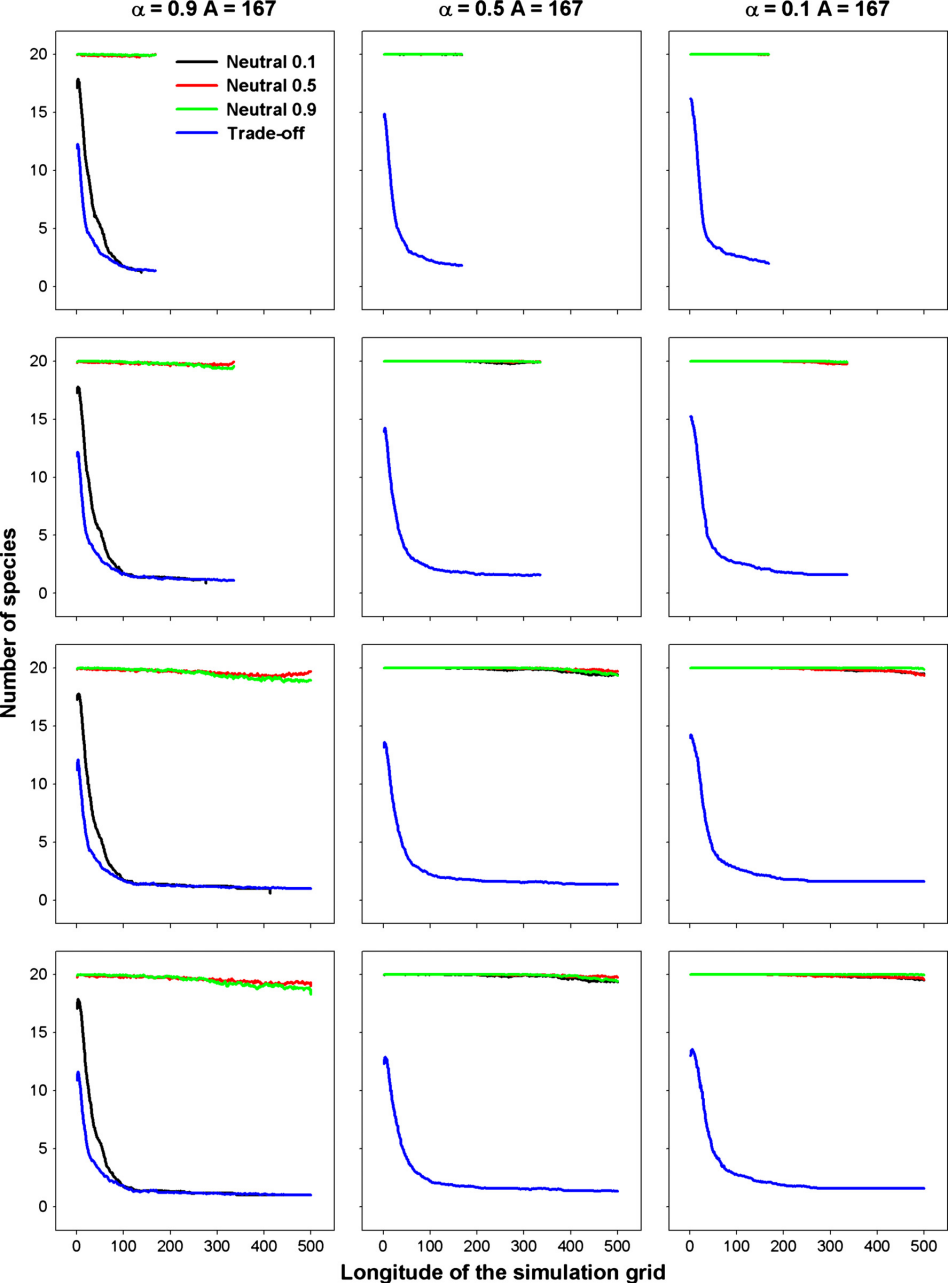
Species richness in communities along the direction of range expansion by the nearest neighbor dispersal under perfect FSTs as compared with those predicted by equal demographics with per capita death rate *d*_*i*_ = 0.1, 0.5, and 0.9, respectively. *α* is a measure of the intensity of dispersal limitation in the nearest neighbor dispersal mode. The rows are for simulations of 1A, 2A, 3A, and 4A years from top to bottom, respectively.

**Figure 2:**
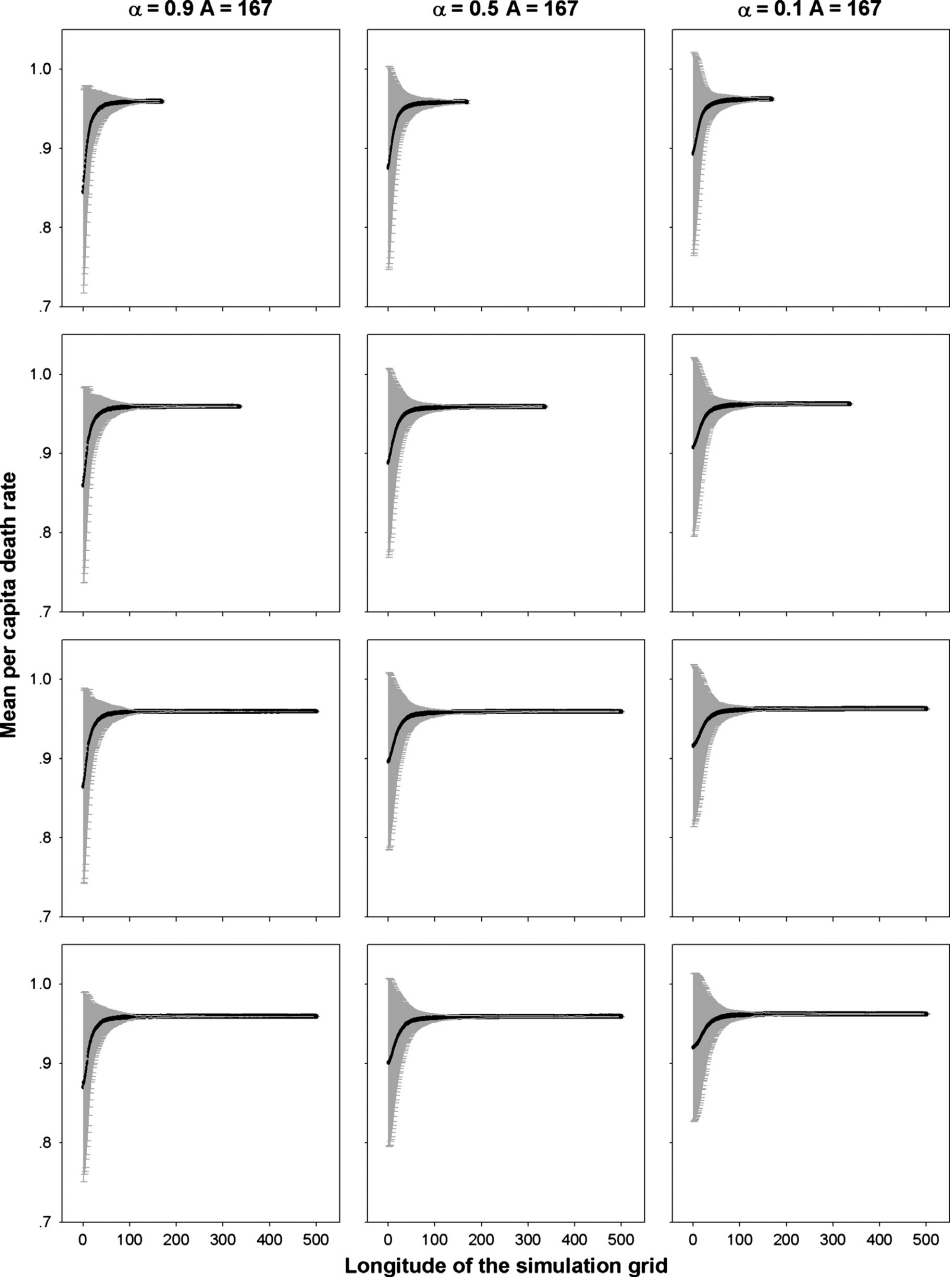
Mean per capita death rates along the longitude of range expansion by the nearest neighbor dispersal in the case of FSTs. Gray areas indicate the mean standard deviations. *α* is a measure of the intensity of dispersal limitation in the nearest neighbor dispersal mode. The rows are for simulations of 1A, 2A, 3A, and 4A years from top to bottom, respectively.

**Figure 3:**
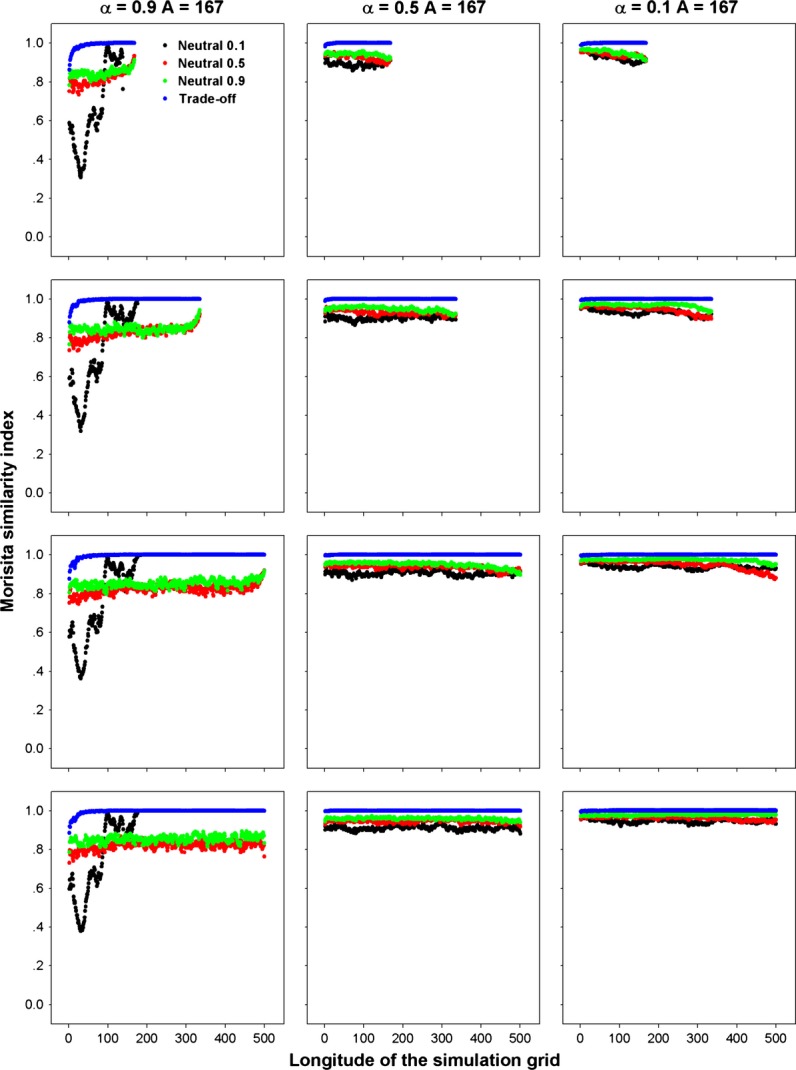
Similarity among communities with the same longitudes generated by the nearest neighbor dispersal measured by Morisita similarity index under perfect FSTs as compared with those predicted by equal demographics with per capita death rate *d*_*i*_ = 0.1, 0.5, and 0.9, respectively. *α* is a measure of the intensity of dispersal limitation in the nearest neighbor dispersal mode. The rows are for simulations of 1A, 2A, 3A, and 4A years from top to bottom, respectively.

Community assembly mechanisms largely affect the species diversity maintained during a range expansion. The results for different species diversity metrics, that is species richness, Simpson's diversity index and Shannon's diversity index, are quite similar; hence, we just report those for species richness as examples. When seed dispersal is restricted within eight nearest neighbor sites, an assemblage under FST demonstrates a steep initial decline and subsequent shallower declines in species richness along the longitude of expansions (Fig. 1). In contrast, a decline in species richness is almost undetectable in strict neutral cases along the expanded ranges, except for a neutral assemblage experiencing a low per capita death rate (*d*_*i*_ = *b*_*i*_ = 0.1) and very strong dispersal limitation (*α* = 0.9) that exhibits a reduction in species richness similar to that under FST (Fig. 1). In the case of a FST, a range expansion acts like a sieve to select those more fecund species, resulting in nearly neutral communities composed of those species with high fecundity/death rates near the wave front (Fig. 2). This also explains the higher similarity among communities of the same longitude under FSTs than those under neutral cases near the wave front (Fig. 3).

**Figure 4:**
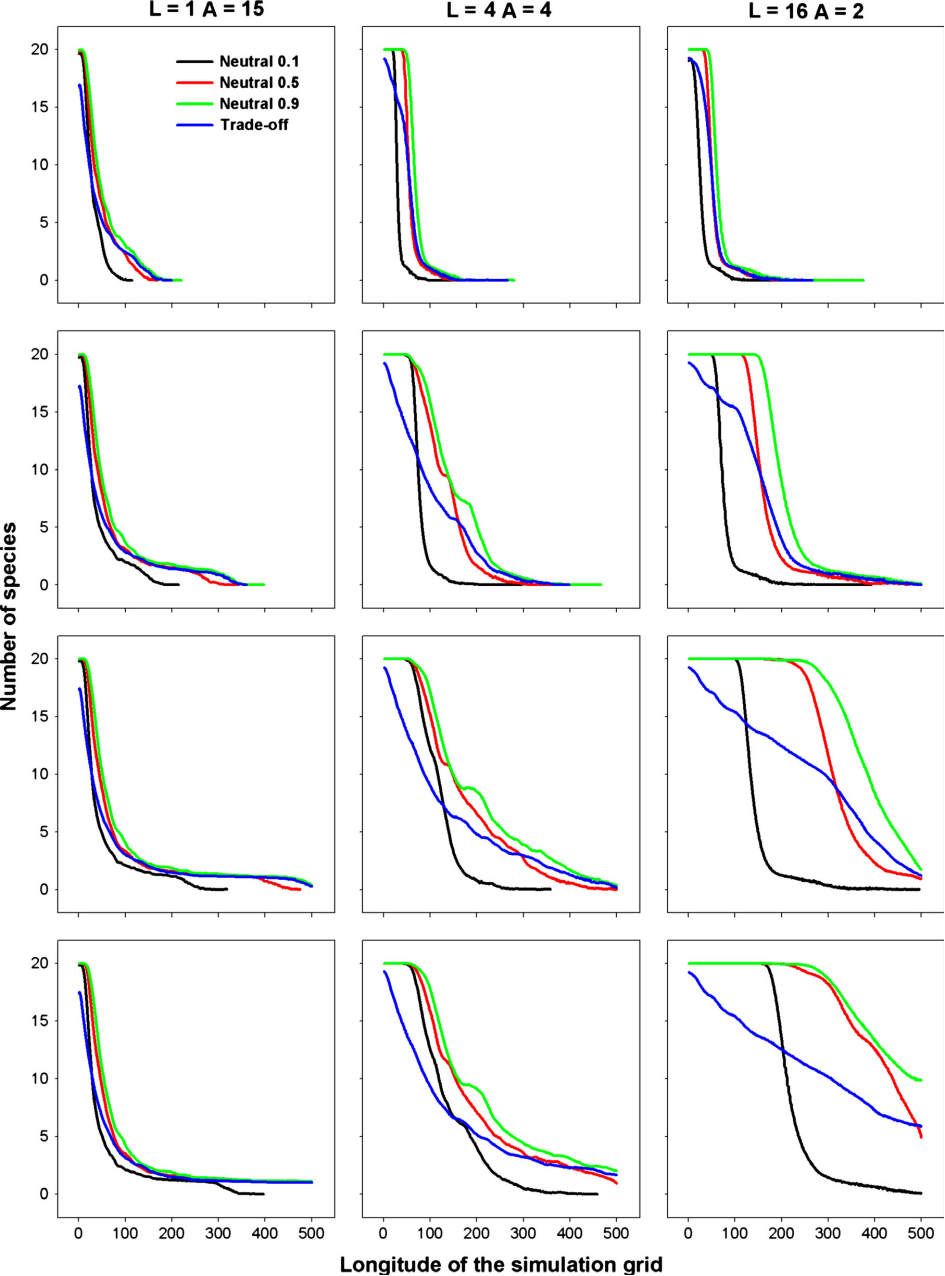
Species richness in communities along the direction of range expansion by a fat-tailed dispersal under perfect FSTs as compared with those predicted by equal demographics with per capita death rate *d*_*i*_ = 0.1, 0.5, and 0.9, respectively. *L* indicates dispersal limitation in the fat-tailed dispersal mode. The rows are for simulations of 1A, 2A, 3A, and 4A years from top to bottom, respectively.

**Figure 5:**
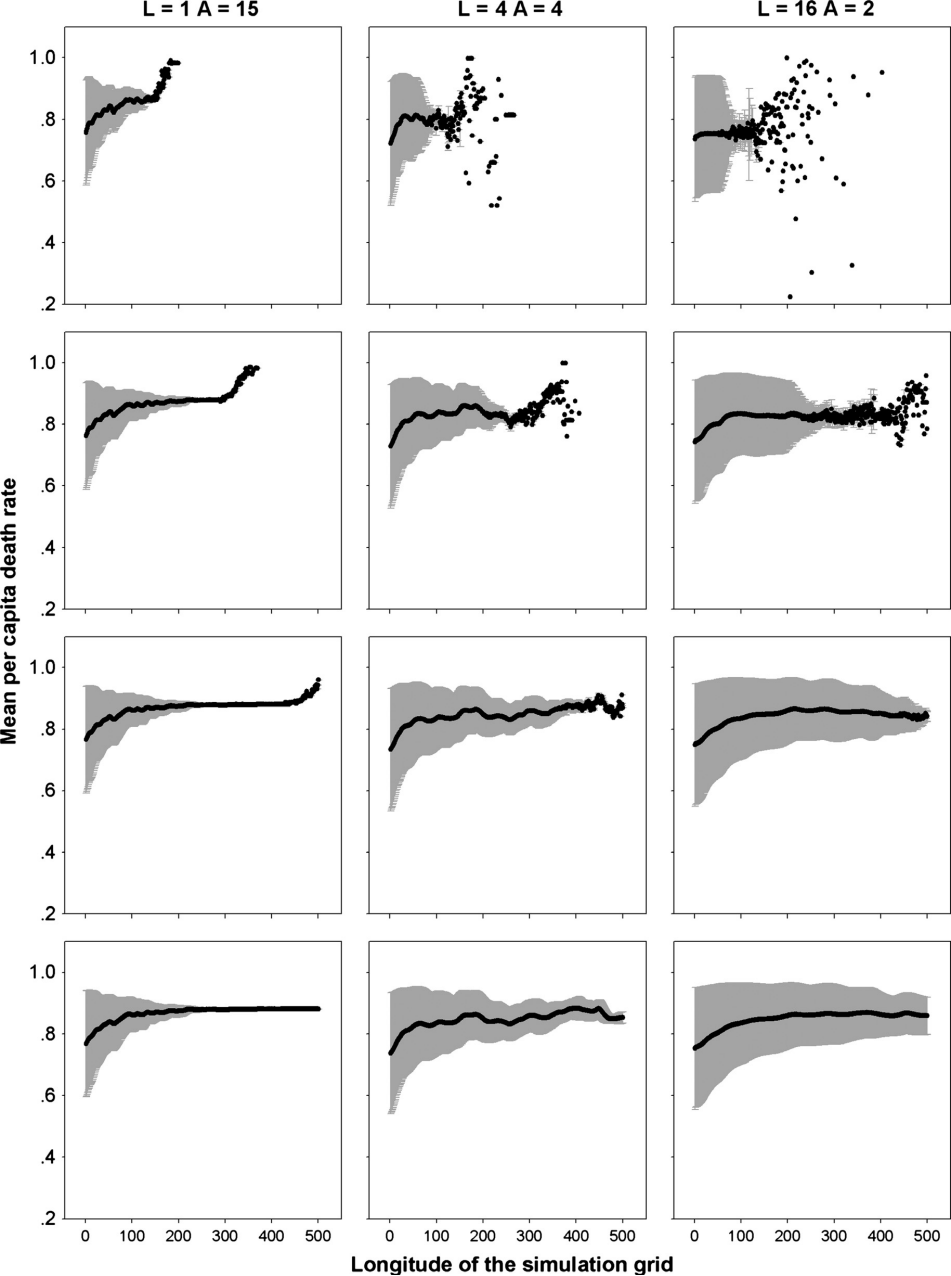
Mean per capita death rates along the longitude of range expansion by a fat-tailed dispersal in the case of FSTs. Gray areas indicate the mean standard deviations. *L* indicates dispersal limitation in the fat-tailed dispersal mode. The rows are for simulations of 1A, 2A, 3A, and 4A years from top to bottom, respectively.

**Figure 6:**
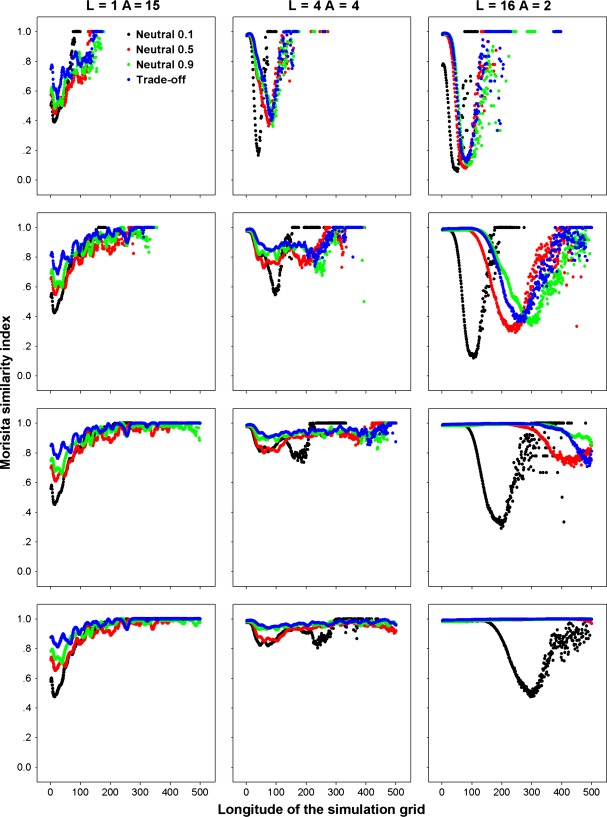
Similarity among communities with the same longitudes generated by a fat-tailed dispersal measured by Morisita similarity index under perfect FSTs as compared with those predicted by equal demographics with per capita death rate *d*_*i*_ = 0.1, 0.5, and 0.9, respectively. L indicates dispersal limitation in the fat-tailed dispersal mode. The rows are for simulations of 1A, 2A, 3A, and 4A years from top to bottom, respectively.

In the case of a fat-tailed dispersal, the erosion patterns of species richness in species assemblages along the expanding direction are similar if dispersal is strongly limited (*L* = 1), but divergent for weak dispersal limitations (*L* = 4, 16), when comparing FST with neutral communities (Fig. 4). In the neutral case, the range expansion of multiple species is governed by a series of successive founder events. Hence, the diversity of the communities near the core community can be rescued by migration from the communities of a higher diversity on their left, while the structure of the communities near the advancing frontier are dominated by the surfing of few species, resulting in declined species richness toward the tip of the wave. A higher per capita fecundity (death) rate can delay the erosion of biodiversity along the expanded range because a higher fecundity also purports more seeds dispersed (higher colonization) for each species. For FSTs under a weak dispersal limitation (*L* = 4, 16), the decline in biodiversity always accompanies the range expansions (Fig. 4). However, the loss of biodiversity under FSTs is governed by both the successive founder effect and competitive exclusion of less fecund species by more fecund ones (Fig. 5). Thus FSTs will result in a lower biodiversity than predicted by the neutral model near the core community, while a higher diversity may be expected near the advancing front depending on the relative mean fecundity rates in a FST and neutral case (Fig. 4). For the same reason as for the nearest neighbor dispersal, communities in the same column share a higher similarity under FSTs than under the neutral model (Fig. 6).

## Discussion

Most previous studies on species range limits have put an asymmetric attention on single or several species, failing to take the whole community into consideration. Here, we showed that, although a decline in species diversity of range expanding communities is expected as a parallel to the decreased genetic diversity of a single species experiencing a range expansion, the neutral model generally predicts a higher biodiversity near the core of the range expansion, and a lower community similarity compared with a FST model. Besides this, an assortment by the reproduction rate may lead to nearly neutral communities near the tip of the wave under FSTs. In the case of FST, given that different species share the same dispersal mode, species with higher fecundities may also possess higher colonization rates because of the larger number of seeds produced and dispersed. Hence, those species with higher fecundity rates will win the race of a range expansion, leading to a decline in biodiversity but an increase in community similarity than predicted by the neutral model at the wave front.

At a single species level, it is widely accepted that assortment by dispersal ability on the expanding front pushes the evolution of increased dispersal (Simmons and Thomas 2004; Hanski et al. 2006; Lindstrom et al. 2013). An alternative explanation for a successful range expansion is the selection for a higher fecundity rate at the advancing front (Neubert and Caswell 2000; Phillips 2009). Using a common garden design, Phillips (2009) showed that both tadpoles and juvenile toads (*Bufo marinus*) from frontal populations would grow about 30% faster than those from older, long established populations of cane toads across northern Australia. He concluded that, because individuals in the advancing front face a lower population density than those in an established population, they may experience a greater r-selection (Phillips 2009). Neubert and Caswell (2000) constructed a discrete-time model accounting for both an individual's vital rate and dispersal rate and found that the sensitivity and elasticity of invasion speed are highly correlated with the sensitivity and elasticity of the population growth rates in two example plants. Theoretical studies also verified the evolution of life history traits during range expansions. For example, Burton et al. (2010) constructed a three-trait trade-off model to explore the evolution of dispersal, reproduction and competitive ability during a range expansion and found that both dispersal and reproduction are selected on the expanding population front.

In contrast to the interest in the evolution of dispersal and reproduction at a given species' advancing front, previous comparisons within plant genera, families or environments have conflicted over the differences in reproductive traits between native and invasive species (Richardson et al. 2000; Buckley et al. 2003; Daehler 2003). Recently, Mason et al. (2008) analyzed the data for plant reproductive traits collected globally regardless of the genus, family, or habitat involved and concluded that neither seed mass nor seed production of an invasive species differed between their introduced and original ranges. Instead, they found that seed production was greater for the invasive species overall and within herb and woody growth forms. For a given seed mass, the invasive species produced several times more seeds per individual per year than the native species. Mason et al. (2008) went further and demonstrated the possibility of assortment by reproduction ability when considering multiple species range expansion simultaneously. This implies that assortment of dispersal and reproduction ability may happen on the wave front without evolution of life history traits, which is consistent with our conclusions. In this study, we show theoretically that even without evolution of traits, a range expansion alone will select for those species possessing higher reproduction rates, although at the expense of a higher death rate or lower competitive ability. This conclusion is also verified in the context of succession, where species with r-strategy (a higher fecundity rate) generally dominate in the early stage of a succession (Chu et al. 2007).

As shown before (Clark 1998), both the shape of the dispersal kernel and the intensity of dispersal limitation largely affect the biodiversity maintained in communities along the expanding gradient. In spite of this, species assemblages regulated by different coexistence rules reveal quite different patterns in the erosion of biodiversity and variation in diversity within the expanded area. These results indicate that communities assembled by different mechanisms may respond to environmental changes in quite different ways, emphasizing the importance of taking community assembly rules into account when considering the effect of global changes on biodiversity.

In this study, the community dynamics within each site is neutral because of the spatially implicit assumption or the mean field assumption (Lin et al. 2009). If dispersal limitation is introduced into the dynamics of communities within sites, species with a higher fecundity rate will dominate the community dynamics within each site, thus a further lower biodiversity and higher community similarity may be expected than we report here for the FST cases.

According to the “principle of allocation” in life history evolution (Stearns 1992), a high fecundity can only be expected at the cost of decreased investment in survival, competition, etc. Hence, we expect that a trade-off between fecundity and survival should be one of the most prevalent life history trade-offs in real communities. The patterns we observed in this study may also apply for a trade-off between colonization and survival because what a species benefit from a high fecundity is actually high colonization rates of vacant sites during range expansion. However, further investigations may be necessary for understanding the influences of other more complex trade-offs on the diversity of expanding communities.

## Supporting Information

**Data S1.** The simulation code, a sample run and the corresponding results.
