# Supplementary material for: Community assembly rules affect the diversity of expanding communities
Source: Ecol Evol. 2014 Oct 3;4(21):4041–52. doi: 10.1002/ece3.1251 (PMC4242558; doi:10.1002/ece3.1251)
Supplement: Supplementary file 1 — Data S1. The simulation code, a sample run and the corresponding results. [file ece30004-4041-SD1.zip › ECE_31251_Running example.docx]

# running example

# One will get simulation results as illustrated in “output example.xls” for the following input parameters:

546 #a random number can avoid pseudo-random number affect

1 # select nearest dispersal

0.5 # the intensity of dispersal limitation in the nearest neighbor dispersal mode

1 # select neutral mode

0.9 #mortality

Output example.xls # output filename

50 # simulate times
